# DEVELOPMENT OF A CLINICAL GUIDELINE FOR FAMILY-CENTERED COLLABORATIVE CARE IN PATIENTS WITH CHRONIC MENTAL DISORDERS

**DOI:** 10.1192/j.eurpsy.2023.1887

**Published:** 2023-07-19

**Authors:** R. Dehbozorgi, M. Fereidooni-Moghadam, M. Shahriari, E. Moghimi-Sarani

**Affiliations:** 1 Nursing and Midwifery School, Isfahan University of Medical Sciences, Isfahan, Iran, Isfahan; 2 Department of Psychiatry Research Center For Psychiatry And Behavioral Science, Shiraz University of Medical Sciences, Shiraz, Iran, Islamic Republic Of

## Abstract

**Introduction: Background:**

Chronic mental illnesses have long periods, are recurring, and require continuous care and, becoming chronic, they double the problems of patients and their family. The study’s purpose is, thus, the development of guidelines for family-centered collaborative care of patients with chronic mental illnesses referring to the medical centers (1).

**Methods:**

This mixed methods study is based on the stages of the guideline adaptation provided by the Guidelines International Network (2).

**Results:**

After reviewing and gathering evidence from a qualitative study on key participants and reviewing the literature, which includes a search for articles related to participatory family-centered care of patients with chronic mental disorders and a review of available books, a clinical guide Related and upstream documents of the country were 531 recommendations were extracted and sent to a panel of experts for evaluation.

**Conclusion:**

Providing a family-centered collaborative care guideline will improve the quality of life of these individuals and their families, improve the quality of care, and reduce fragmented health care(3,4).

**Objectives:**

Development of a clinical guideline for family-centered collaborative care in patients with chronic mental disorders**Methods:** This mixed methods study is based on the stages of the guideline adaptation provided by the Guidelines International Network .

**Results:**

After reviewing and gathering evidence from a qualitative study on key participants and reviewing the literature, which includes a search for articles related to participatory family-centered care of patients with chronic mental disorders and a review of available books, a clinical guide Related and upstream documents of the country were 531 recommendations were extracted and sent to a panel of experts for evaluation.

**Conclusions:**

Providing a family-centered collaborative care guideline will improve the quality of life of these individuals and their families, improve the quality of care, and reduce fragmented health care

**Reference(s):**

1) Vigo D, Thornicroft G, Atun R. 2016*,Estimating the true global burden of mental illness.* The Lancet Psychiatry.3(2):171-8.

2) GIN. 2010, *guideline Adaptation a source toolkit* guideline international network; 95

3) Molu NG, Ozkan B, Icel S. 2016*,Quality of life for chronic psychiatric illnesses and home care.* Pakistan journal of medical sciences.32(2):511-5

4) WHO. 2014, *Integrating the response to mental disorders and other chronic diseases in health care systems.* Geneva, Switzerland: World Health Organization; 50 p

**Disclosure of Interest:**

None Declared

